# Smyd1b_tv1, a Key Regulator of Sarcomere Assembly, Is Localized on the M-Line of Skeletal Muscle Fibers

**DOI:** 10.1371/journal.pone.0028524

**Published:** 2011-12-09

**Authors:** Huiqing Li, Jin Xu, Yue-Hong Bian, Pep Rotllant, Tiansheng Shen, Wuying Chu, Jianshe Zhang, Martin Schneider, Shao Jun Du

**Affiliations:** 1 Department of Biochemistry and Molecular Biology, University of Maryland School of Medicine, Baltimore, Maryland, United States of America; 2 Department of Bioengeneering and Environmental Science, Changsha University, Hunan, China; Consejo Superior de Investigaciones Cientificas, Spain

## Abstract

**Background:**

Smyd1b is a member of the Smyd family that plays a key role in sarcomere assembly during myofibrillogenesis. *Smyd1b* encodes two alternatively spliced isoforms, *smyd1b_tv1* and *smyd1b_tv2*, that are expressed in skeletal and cardiac muscles and play a vital role in myofibrillogenesis in skeletal muscles of zebrafish embryos.

**Methodology/Principal Findings:**

To better understand Smyd1b function in myofibrillogenesis, we analyzed the subcellular localization of Smyd1b_tv1 and Smyd1b_tv2 in transgenic zebrafish expressing a myc-tagged Smyd1b_tv1 or Smyd1b_tv2. The results showed a dynamic change of their subcellular localization during muscle cell differentiation. Smyd1b_tv1 and Smyd1b_tv2 were primarily localized in the cytosol of myoblasts and myotubes at early stage zebrafish embryos. However, in mature myofibers, Smyd1b_tv1, and to a small degree of Smyd1b_tv2, exhibited a sarcomeric localization. Double staining with sarcomeric markers revealed that Smyd1b_tv1was localized on the M-lines. The sarcomeric localization was confirmed in zebrafish embryos expressing the Smyd1b_tv1-GFP or Smyd1b_tv2-GFP fusion proteins. Compared with Smyd1b_tv1, Smyd1b_tv2, however, showed a weak sarcomeric localization. Smyd1b_tv1 differs from Smyd1b_tv2 by a 13 amino acid insertion encoded by exon 5, suggesting that some residues within the 13 aa insertion may be critical for the strong sarcomeric localization of Smyd1b_tv1. Sequence comparison with Smyd1b_tv1 orthologs from other vertebrates revealed several highly conserved residues (Phe223, His224 and Gln226) and two potential phosphorylation sites (Thr221 and Ser225) within the 13 aa insertion. To determine whether these residues are involved in the increased sarcomeric localization of Smyd1b_tv1, we mutated these residues into alanine. Substitution of Phe223 or Ser225 with alanine significantly reduced the sarcomeric localization of Smyd1b_tv1. In contrast, other substitutions had no effect. Moreover, replacing Ser225 with threonine (S225T) retained the strong sarcomeric localization of Smyd1b_tv1.

**Conclusion/Significance:**

Together, these data indicate that Phe223 and Ser225 are required for the M-line localization of Smyd1b_tv1.

## Introduction

Smyd1, also known as Bop, is a member of the Smyd family that plays a key role in muscle cell differentiation [1]–[4]. *Smyd1* encodes two alternatively spliced isoforms, *smyd1_tv1* and *smyd1_tv2*, that are expressed in skeletal and cardiac muscles [3], [4]. Smyd1_tv1 differs from Smyd1_tv2 by containing a 13 amino acid insertion encoded by the *smyd1_tv1*-specific exon 5 [3], [4]. Targeted deletion of *smyd1* in mice resulted in defective ventricle formation and early embryonic lethality at E10.5, suggesting a vital role of Smyd1 in cardiomyogenesis [3]. Knockdown of *smyd1b* expression in zebrafish resulted in paralyzed zebrafish larvae with defective myofibril assembly in skeletal myofibers [4].

The molecular mechanism by which Smyd1 controls the myofibrillogenesis is not clear. Biochemical studies indicate that Smyd1b could methylate histone H3 proteins *in vitro* [4]. Consistent with its potential function in transcriptional regulation, Smyd1 is initially localized in the nucleus of C2C12 myoblasts [5]. *In vitro* studies have revealed that *smyd1* represses gene transcription in a histone deacetylase (HDAC) dependent fashion [3]. However, it has been reported that Smyd1 undergoes a nucleus to cytoplasm translocation during myoblast differentiation into myotubes [5], suggesting that Smyd1 may have additional function in the cytoplasm.

A better characterization of Smyd1b localization is critical for the mechanistic understanding of Smyd1b function in regulating muscle cell differentiation. In this study, we analyzed the subcellular localization Smyd1b_tv1 and Smyd1b_tv2 during muscle development in zebrafish embryos as well as in adult skeletal muscles. The data showed that Smyd1b_tv1 and Smyd1b_tv2 were primarily localized in the cytosol of myoblasts and myotubes of zebrafish embryos at the early stage. However, in mature myofibers of late stage embryos, a sarcomeric localization was evident for Smyd1b_tv1 and Smyd1b_tv2 although Smyd1b_tv2 appeared to be weaker. Double immunostaining with M- or Z-line markers revealed that Smyd1b_tv1 was localized on the M-line of sarcomeres. The strong M-line localization requires Phe223 and Ser225 located within the Smyd1b_tv1-specific 13 aa insertion. Mutation of Phe223 or Ser225 to alanine significantly reduced the sarcomeric localization of Smyd1b_tv1. In contrast, replacing Ser225 with threonine had no effect on the Smyd1b_tv1 sarcomeric localization

## Results

### Characterization of Smyd1b_tv1 and Smyd1b_tv2 subcellular localization during muscle development in zebrafish embryos

**Figure 1:**
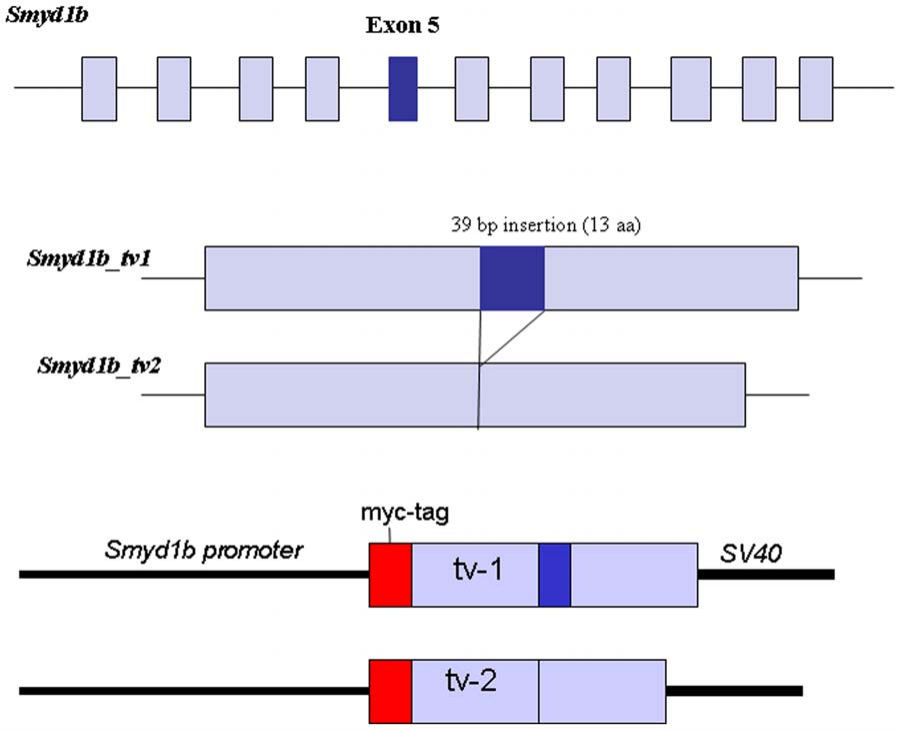
Generation of Smyd1b_tv1 and Smyd1b_tv2 by alternative splicing and construction of the Smyd1b_tv1^myc^ and Smyd1b_tv2^myc^ transgenes. *Smyd1b_tv1* and *Smyd1b_tv2* transcripts are generated by alternative splicing. Their cDNA sequences are identical, with the exception of the 39 bp insertion encoded by exon 5. It translates into a 13 amino acids insertion in Smyd1b_tv1. Smyd1b_tv1^myc^ and Smyd1b_tv2^myc^ transgenes are constructed by fusing with an in frame myc-tag at the N-terminus. Expression of the transgenes are directed by its own promoter. A SV40 PolyA signal was included at the 3′ end.

**Figure 2:**
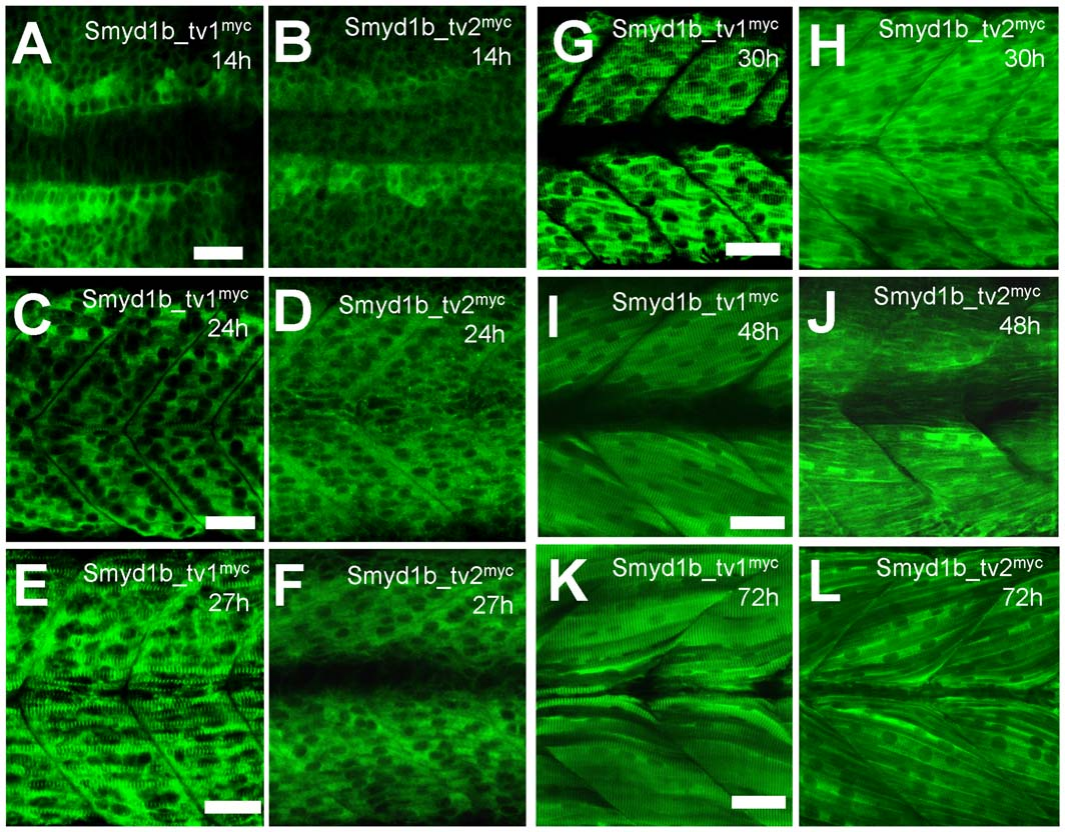
Smyd1b_tv1^myc^ and Smyd1b_tv2^myc^ show dynamic localizations during muscle cell differentiation in zebrafish embryos. **A–D.** Whole-mount immunostaining with anti-myc antibody shows the cytosolic localization of Smyd1b_tv1^myc^ (A, C) or Smyd1b_tv2^myc^ (B, D) in myoblasts of transgenic zebrafish embryos at 14 and 24 hours-post-fertilization (hpf), respectively. A and B, dorsal views. C and D, side views. **E, G, I, K.** Immunostaining with anti-myc antibody shows the sarcomeric localization of Smyd1b_tv1^myc^ in myofibers of transgenic fish embryos at 27, 30, 48, and 72 hpf, respectively. **F, H, J, L.** Immunostaining with anti-myc antibody shows the cytosolic (F, H, J) and sarcomeric (L) localization of Smyd1b_tv2^myc^ in myofibers of transgenic fish embryos at 27, 30, 48, and 72 hpf, respectively. Scale bars: 30 µm.

Previous studies have shown that Smyd1 undergoes a nucleus to cytoplasm translocation during C2C12 myoblast differentiation *in vitro* [5]. It is not clear whether the two isoforms, Smyd1b_tv1 and Smyd1b_tv2, from alternative splicing have similar or distinct subcellular localization in muscle cells during muscle development. To better understand Smyd1b function in myofibril assembly, we analyzed the subcellular localization of Smyd1b_tv1 and Smyd1b_tv2 during muscle development using transgenic zebrafish models that expressed a myc-tagged Smyd1b_tv1 or Smyd1b_tv2 under the control of its own promoter (Fig. 1). The results showed a dynamic subcellular localization of Smyd1b_tv1^myc^ and Smyd1b_tv2^myc^ during muscle development. In early stage embryos at 14 and 24 hpf, Smyd1b_tv1 and Smyd1b_tv2 were primarily localized in the cytosol of myoblast and myotubes with little or no nuclear localization (Fig. 2A–D). However, as embryos develop into late stages, a clear sarcomeric localization was detected for Smyd1b_tv1 in differentiated myofibers at 27 hpf (Fig. 2E). The sarcomeric localization appeared earlier for Smyd1b_tv1 than Smyd1b_tv2 (Fig. 2F). The sarcomeric localization of Smyd1b_tv1^myc^ was maintained throughout early development (Fig. 2G, I, K). In contrast, a weak sarcomeric localization of Smyd1b_tv2 could be detected in zebrafish embryos at 72 hpf (Fig. 2L).

**Figure 3:**
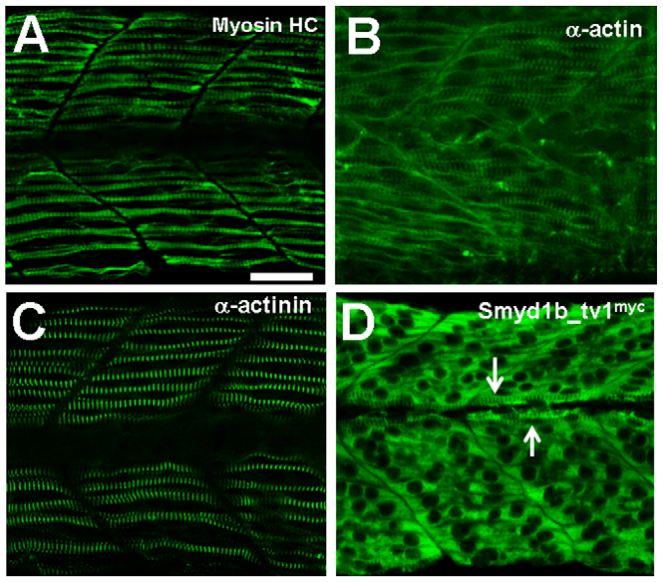
The sarcomeric localization of Smyd1b_tv1 occurs after the sarcomere formation in myofibers of zebrafish embryos. **A–C.** Immunostaining using sarcomeric specific antibodies against MyHC (A), α-actin (B), and α-actinin (C) in the trunk muscles of zebrafish embryos at 24 hpf. **D.** Immunostaining using anti-myc antibody shows the primary cytoplasmic localization of Smyd1b_tv1 in the trunk muscles of *smyd1b_tv1^myc^* transgenic fish embryos at 24 hpf. Muscle pioneer cells with the sarcomeric localization are indicated by arrows. Scale bar: 30 µm.

The timing of Smyd1b_tv1^myc^ sarcomeric localization was compared with other sarcomeric proteins in trunk muscles of the same stage zebrafish embryos. The results showed that the sarcomeric localization of myosin heavy chain, α-actin and α-actinin occurred before the Smyd1b_tv1 sarcomeric localization (Fig. 3). Thick and thin filaments as well as Z-lines were clearly organized in myofibers of zebrafish embryos at 24 hpf (Fig. 3A–C). In contrast, there was little sarcomeric localization of Smyd1b-tv1^myc^ in trunk muscles of the same stage embryos, except muscle pioneer cells in the myoseptum region representing the first group of muscle cells to differentiate in zebrafish embryos (Fig. 3D). Collectively, these data indicate that the sarcomeric localization of Smyd1b_tv1 occurred after that of myosin, α-actin and α-actinin, suggesting that although Smyd1b is required for myofibril assembly, the sarcomeric localization of Smyd1b_tv1 was not required in this initial process.

### Characterization of Smyd1b_tv1 and Smyd1b_tv2 subcellular localization using GFP fusion proteins

**Figure 4:**
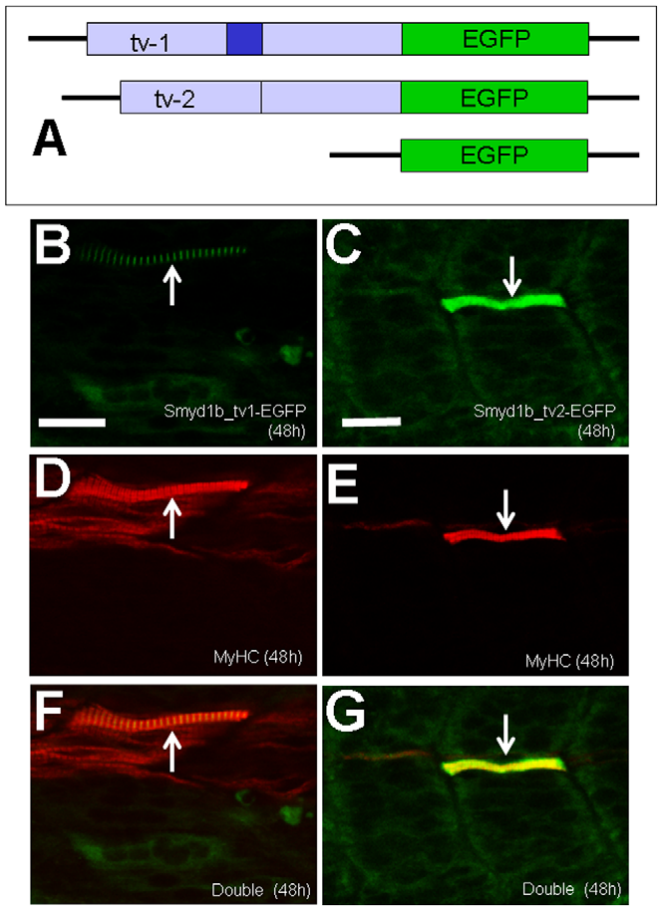
Rescue of myofibril organization defect in smyd1b knockdown embryos by expression of Smyd1b_tv1-EGFP or Smyd1b_tv2-EGFP fusion protein. **A.** DNA constructs encoding Smyd1b_tv1-EGFP, or Smyd1b_tv2-EGFP fusion proteins or EGFP control were generated and injected into zebrafish embryos. **B and C.** Myofibers expressing Smyd1_tv1-EGFP (B) or Smyd1_tv2-EGFP (C) was directly observed by GFP. **D and E.** Myosin thick filaments organization was determined by F59 antibody staining in Smyd1_tv1-EGFP (D) or Smyd1_tv2-EGFP (E) co-injected embryos. **F and G.** Double staining shows the colocalization of normal fibers with Smyd1_tv1-EGFP (F) or Smyd1_tv2-EGFP (G) expression. Scale bars: 20 µm.

To better follow the dynamic localization of Smyd1b_tv1 and _tv2 during embryonic muscle development, we generated two DNA constructs, pTol2-smyd1b_tv1-EGFP and pTol2-smyd1b_tv2-EGFP, that express the GFP tagged Smyd1b_tv1 and Smyd1b_tv2 fusion proteins, respectively (Fig. 4A). Rescue assay by co-injecting Smyd1b morpholino (MO) with pTol2-smyd1b_tv1-EGFP or pTol2-smyd1_tv2-EGFP construct revealed that Smyd1b_tv1-EGFP and Smyd1b_tv2-EGFP fusion proteins are biologically active. Both Smyd1b_tv1-EGFP and Smyd1_tv2-EGFP could rescue the myofibril defects from Smyd1b knockdown (Fig. 4B–G). Expression of Smyd1b_tv1-EGFP or Smyd1_tv2-EGFP resulted in a mosaic pattern of normal myofibers in 90% (n = 60) of *smyd1b* knockdown embryos (Fig. 4F, G). In contrast, embryos co-injected with the Smyd1b MO and EGFP vector control showed no normal myofibers (data not shown). Similar to myc-tagged proteins, a sarcomeric localization was detected for Smyd1b_tv1-EGFP but not for Smyd1_tv2-EGFP (Fig. 4B, C). Collectively, these data indicate that Smyd1b_tv1-EGFP and Smyd1b_tv2-EGFP fusion proteins are biologically active and exhibit similar localization with the respective myc-tagged proteins.

**Figure 5:**
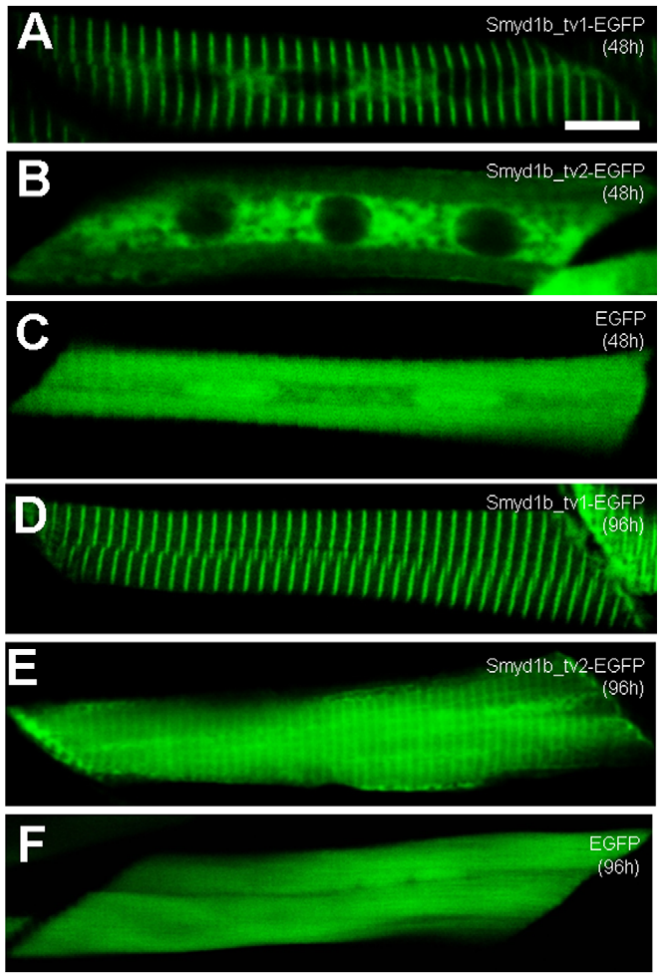
Characterization of the sarcomeric localization using Smyd1b_tv1-EGFP and Smyd1b_tv2-EGFP fusion proteins. DNA constructs encoding Smyd1b_tv1-EGFP, or Smyd1b_tv2-EGFP fusion proteins or EGFP control injected into zebrafish embryos. Their expression and localization was determined in myofibers of the injected zebrafish embryos at 48 (A–C) and 96 (D–F) hpf. A and D, Smyd1b_tv1-EGFP; B and E, Smyd1b_tv2-EGFP; C and F, EGFP control. Scale bar: 8 µm.

The expression and subcellular localization of Smyd1b_tv1-EGFP and Smyd1_tv2-EGFP were further characterized in zebrafish embryos at 48 and 96 hpf. Consistent with the data from the myc-tagged proteins, Smyd1b_tv1-EGFP was mainly localized on the sarcomeres of skeletal muscles at 48 and 96 hpf (Fig. 5A, D). In contrast, Smyd1b_tv2-EGFP (Fig. 5B), like the GFP control (Fig. 5C), showed little or no sarcomeric localization in skeletal myofibers of zebrafish embryos at 48 hpf. However, a weak sarcomeric localization was observed with Smyd1b_tv2-EGFP in 96 hpf embryos (Fig. 5E). Together, these studies suggest a dynamic subcellular localization of Smyd1b_tv1 and tv_2 during muscle development.

### Smyd1b_tv1 is localized on the M-line of sarcomeres

**Figure 6:**
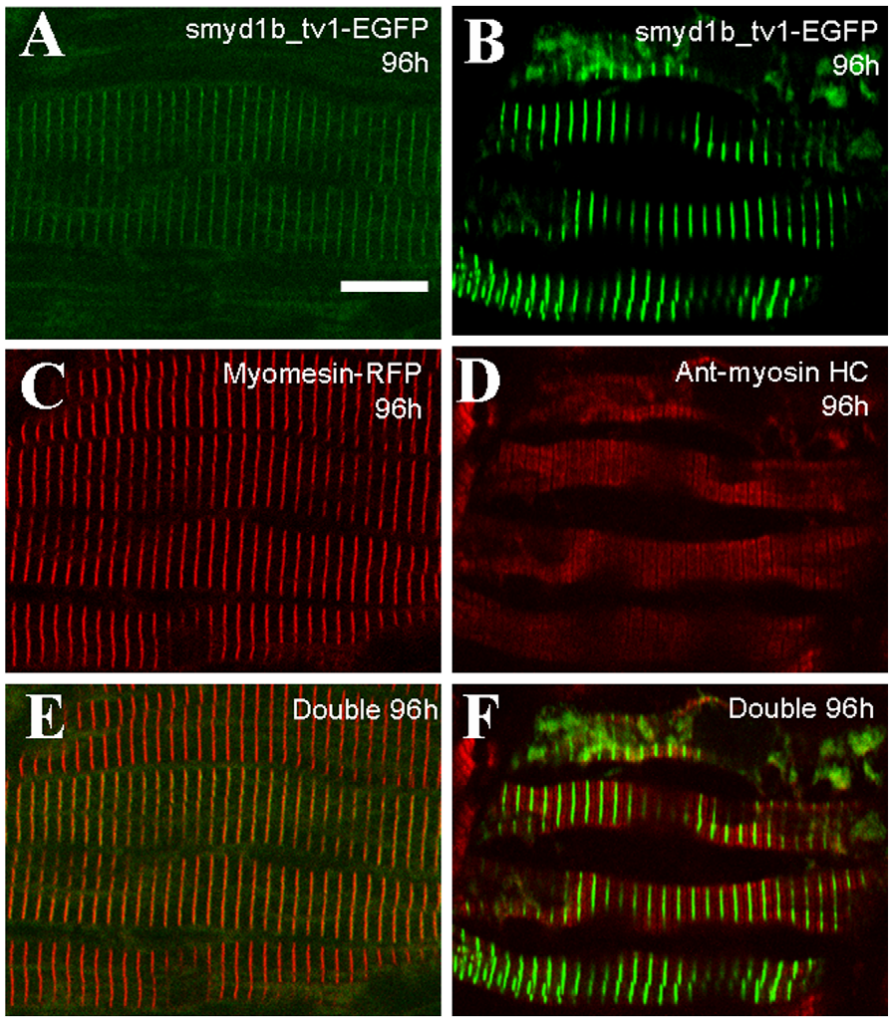
Smyd1b_tv1-EGFP is localized on the M-line of zebrafish skeletal muscles. Smyd1b_tv1-EGFP construct was injected into Myomesin-RFP (A, C, E) or wild type (B, D, F) zebrafish embryos at 1–2 cell stages. Smyd1b_tv1-EGFP localization was determined together with M-line marker (Myomesin-RFP) and A-band marker (Myosin heavy chain) at 96 hpf. **A, B and C.** Co-localization of Smyd1b_tv1-EGFP and Myomesin-RFP was observed in myofibers of the injected embryos. **D, E and F.** Immunostaining with anti-MyHC antibody (F59) shows the localization of Smyd1b_tv1-EGFP in the middle of the A-bands in myofibers of zebrafish embryos. Scale bar: 12 µm.

To better define the sarcomeric localization of Smyd1b_tv1 and _tv2, we localized Smyd1b_tv1-EGFP in a zebrafish embryos expressing a myomesin-RFP fusion protein at the M-line. The pTol2-smyd1b_tv1-EGFP construct was microinjected into the myomesin-RFP zebrafish embryos at 1–2 cells stages. The sarcomeric localization of Smyd1b_tv1-EGFP (green) and myomesin-RFP (red) was determined by confocal microscopy at 96 hpf. A clear co-localization of Smyd1b_tv1-EGFP and myomesin-RFP was observed in myofibers expressing the Smyd1b_tv1-EGFP fusion protein in zebrafish embryos (Fig. 6A, C, E). Moreover, co-staining with anti-MyHC antibody revealed that Smyd1b_tv1-GFP was localized in the middle of the A-band (Fig. 6B, D, F), consistent with the M-line localization of Smyd1b_tv1.

**Figure 7:**
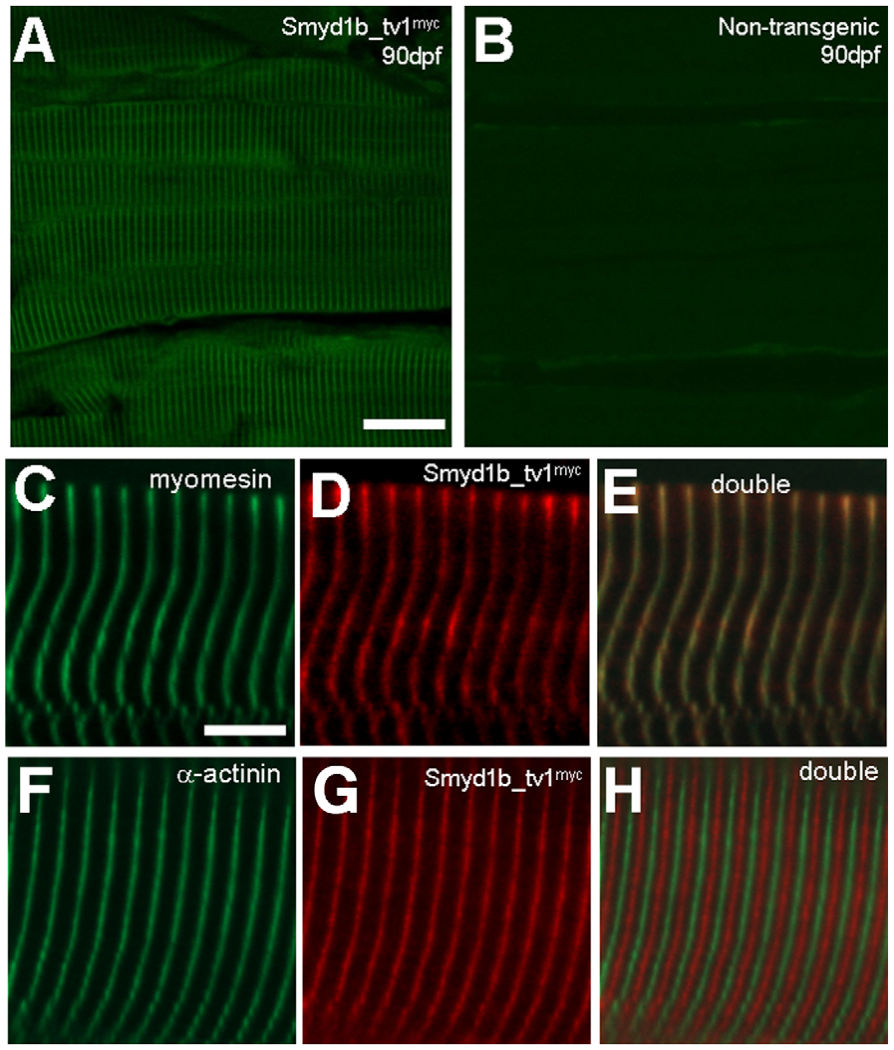
Smyd1b_tv1^myc^ is localized on the M-line of adult zebrafish skeletal muscles. **A and B.** Immunostaining using anti-myc antibody shows the sarcomeric localization of *Smyd1b_tv1^myc^* on longitudinal sections of skeletal muscles from adult transgenic zebrafish expressing a myc-tagged Smyd1b_tv1 (A) or non-transgenic control (B). **C–E.** Double immunostaining with anti-myomesin and anti-myc antibodies shows the colocalization of Smy1b_tv1^myc^ with myomesin on the M-lines. **F–H.** Double immunostaining with anti-α-actinin and anti-myc antibodies shows the non-overlapping localization of Smy1b_tv1^myc^ with α- actinin. Scale bars: A = 22 µm. C = 6 µm.

To determine whether the sarcomeric localization of *smyd1b_tv1^myc^* is maintained in adult skeletal muscles, we carried out an anti-myc antibody staining on adult skeletal muscles of transgenic zebrafish expressing the myc-tagged Smyd1b_tv1^myc^. A clear sarcomeric localization was detected for Smyd1b_tv1^myc^ (Fig. 7A). Double staining with the M-line (myomesin) and Z-line (α-actinin) specific antibodies further confirmed that Smyd1b_tv1^myc^ was co-localized with myomesin on the M-line (Fig. 7E), but not with α-actinin on the Z-line in adult skeletal muscles (Fig. 7H). The M-line localization is consistent with the results from the embryonic muscles. Together, these data indicate that Smyd1b_tv1 is localized on the M-lines of skeletal muscles, and may be involved in M-line organization.

### The enhanced sarcomeric localization of Smyd1b_tv1 requires Phe223 and Ser225 within the Smyd1b_tv1-specific 13 aa insertion

**Figure 8:**
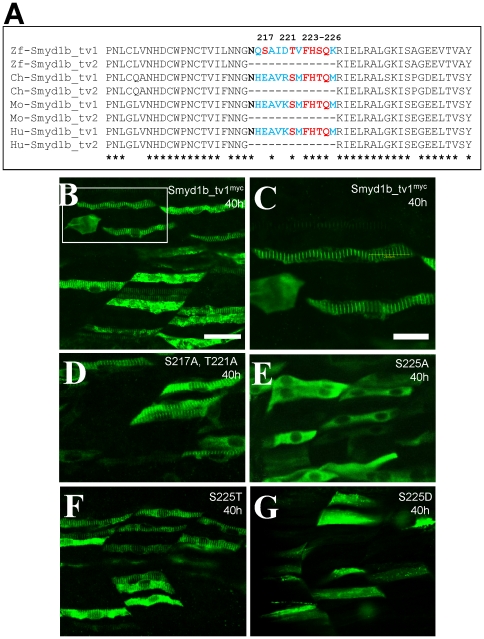
The Serine 225 is required for the enhanced sarcomeric localization of Smyd1b_tv1. **A.** Sequence comparison shows that the alternative splicing of smyd1b in various vertebrates and the conserved serine and threonine residues within the 13 aa insertion. **B and C.** Immunostaining using anti-myc antibody shows the sarcomeric localization of Smyd1b_tv1^myc^ in myofibers of zebrafish embryos at 38 hpf. C represents the highlighted box area in A. **D–G.** Immunostaining using anti-myc antibody shows the sarcomeric localization of Smyd1b_tv1^myc^ mutant proteins that carry substitutions at S217A and T221A (D), S225A (E), S225T (F), S225D (G). Scale bars: B = 40 µm; C = 20 µm.

Compared with Smyd1b_tv2, the stronger sarcomeric localization of Smyd1b_tv1 suggested that the 13 amino acid insertion in the Smyd1b_tv1 might contribute to its increased sarcomeric localization. To identify the key amino acid residue(s) required for the enhanced sarcomeric localization, we compared the protein sequence within the 13 aa insertion among Smyd1b_tv1 orthologs from several vertebrate species. Several potential phosphorylation sites at Ser217, Thr221 and Ser225, were identified (Fig. 8A). To test directly whether these three Ser/Thr residues are required for the sarcomeric localization of Smyd1b_tv1, substitutions were made at these three positions by replacing them with alanine. The mutant proteins were expressed in zebrafish embryos by DNA microinjection. The subcellular localization of mutant proteins was carefully examined in the injected zebrafish embryos by antibody staining. Compared with the control (Fig. 8B, C), substitution of Ser217 and Thr221 with alanine had no effect on the sarcomeric localization of Smyd1b_tv1^myc^ (Fig. 8D). However, substitution of Ser225 with alanine (S225A) abolished the sarcomeric localization of Smyd1b_tv1^myc^ (Fig. 8E). Together, these results indicate that Ser225 is required for the enhanced sarcomeric localization of Smyd1b_tv1.

Sequence comparison revealed that Ser225 was replaced by threonine in Smyd1b_tv1 from chick, mouse and human (Fig. 8A). Serine and threonine are similar type of amino acids that are potential sites for post-translational modification by phosphorylation or glycosylation. To determine whether substitution of S225 with threonine (S225T) had an effect on its sarcomeric localization, we generated the S225T mutant and analyzed the sarcomeric localization of S225T in zebrafish embryos. The results showed that Ser225T substitution had no effect on the sarcomeric localization of Smyd1b_tv1 (Fig. 8F). To determine whether potential phosphorylation of Ser225 could be involved in sarcomeric localization of Smyd1b_tv1, we substituted Ser225 with aspartic acid (S225D). It has been reported that substitution of serine residues with aspartic acid mimics serine phosphorylation [6], [7]. Our results showed that S225D substitution abolished the Smyd1b_tv1 sarcomeric localization (Fig. 8G), indicating that post-translational modification by phosphorylation may not be involved in the increased sarcomeric localization of Smyd1b_tv1.

**Figure 9:**
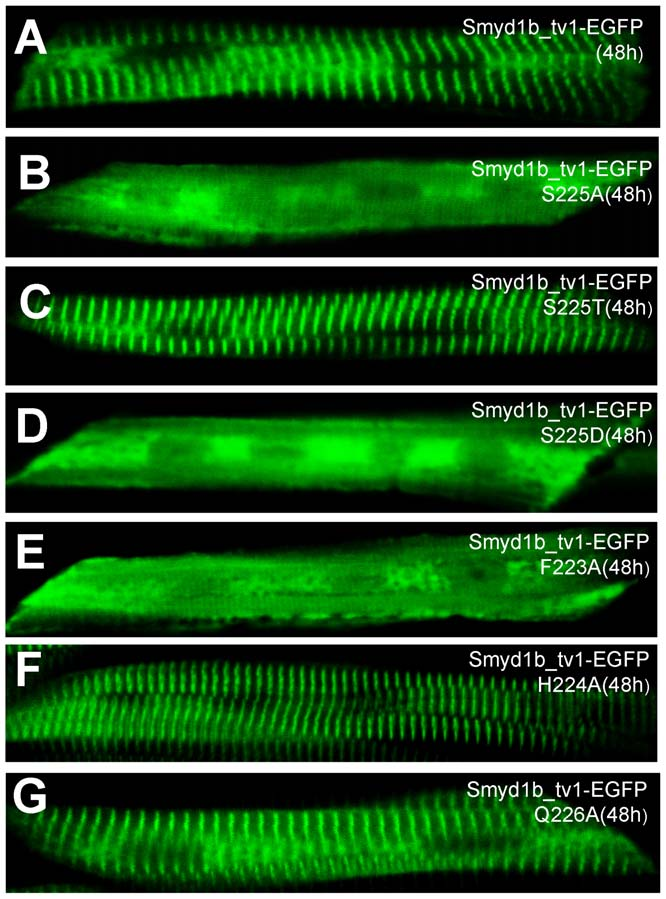
Effect of S225A, S225T, S225D, F223A, H224A and Q226A substitution on the sarcomeric localization of Smyd1_tv1-EGFP in zebrafish embryos. DNA construct expressing Smyd1_tv1-EGFP or its derived mutants of S225A, S225T, S225D, F223A, H224A and Q226A was injected into zebrafish embryos. Their localization was analyzed in myofibers of the injected embryos at 48 hpf. A, Smyd1_tv1-EGFP; B, Smyd1_tv1-EGFP(S225A); C, Smyd1_tv1-EGFP(S225T); D, Smyd1_tv1-EGFP(S225D), E, Smyd1_tv1-EGFP(F223A), F, Smyd1_tv1-EGFP(H224A), and Smyd1_tv1-EGFP(Q226A).

To confirm the data from the myc-tagged Smyd1b mutant proteins, we generated DNA constructs expressing Smy1b_tv1-EGFP fusion proteins with the S225A, S225T or S225D substitutions, and determined the effects on their sarcomeric localization in zebrafish embryos. The data showed that S225A substitution significantly reduced the sarcomeric localization of Smyd1b_tv1-EGFP (S225A) fusion protein (Fig. 9B). In contrast, S225T mutation had little or no effect on the sarcomeric localization of Smyd1b_tv1-EGFP (S225T) (Fig. 9C). Moreover, S225D mutation dramatically reduced the sarcomeric localization of Smyd1b_tv1-EGFP (S225D) fusion protein (Fig. 9D). These data reinforce the idea that the S225 residue is critical for the enhanced sarcomeric localization of Smyd1b_tv1. However, because the phosphomimetic Smyd1b variant (S225D) also leads to abolished M-line translocation, phosphorylation of these amino acids cannot be the cause for the M-line translocation. The S225A substitution might lead to a different protein shape masking or changing the translocation signal required for M-line localization. To evaluate the possibility that the highly conserved amino acids surrounding the Ser225 might be involved in M-line localization, we carried out additional mutations at F223, H224 and Q226. The data showed that mutating the conserved Phe223 (F223) dramatically diminished the M-line localization (Fig. 9E). In contrast, mutating two other conserved residues (H224, Q226) had no effect (Fig. 9F, G). Collectively, the data indicate that Phe223 and Ser225 are required for the sarcomeric localization. S225A or F223A substitution might lead to a different protein shape masking or changing the translocation signal or protein motif required for M-line localization.

### Knockdown of endogenous Smyd1b advances the timing of Smyd1b_tv1^myc^ sarcomeric localization in zebrafish embryos

**Figure 10:**
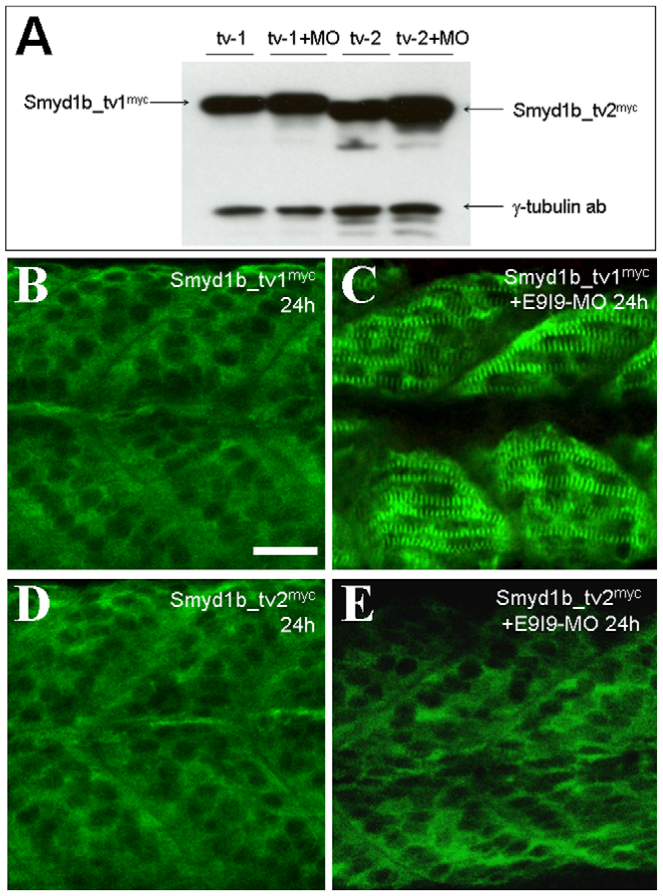
Knockdown of endogenous Smyd1b advances the timing of sarcomeric localization of Smyd1b_tv1^myc^ in zebrafish embryos. **A.** Smyd1b E9I9-MO was injected into Smyd1b_tv1^myc^ or Smyd1b_tv1^myc^ transgenic zebrafish embryos at 1–2 cell stages. Western blot analysis shows the expression of myc-tagged Smyd1b_tv1^myc^ and Smyd1b_tv1^myc^ in un-injected control or E9I9-MO injected transgenic zebrafish embryos at 24 hpf. γ-Tubulin was used as loading control. **B and C.** Immunostaining using anti-myc antibody shows the cytoplasmic (B) or sarcomeric localization (C) of *smyd1b_tv1^myc^* in control (B) or E9I9-MO injected (C) transgenic zebrafish embryos at 24 hpf. **D and E.** Immunostaining using anti-myc antibody shows the cytoplasmic localization of *smyd1b_tv2^myc^* in control or E9I9-MO injected transgenic zebrafish embryos at 24 hpf. Scale bar: 30 µm.

To determine whether knockdown of endogenous Smyd1b expression could affect the expression and sarcomeric localization of Smyd1b_tv1^myc^ and Smyd1b_tv2^myc^ from the transgene, the splicing MO (E9I9-MO) was injected into *smyd1b_tv1* transgenic zebrafish embryos expressing the Smyd1b_tv1^myc^ or Smyd1b_tv2^myc^ fusion proteins. Western blot analysis revealed that the E9I9-MO could not knock down the expression of the myc-tagged smyd1b_tv1or smyd1b_tv2 from the transgene because they were constructed using the cDNA coding sequence and the expression does not require RNA splicing. Compared with the uninjected respective transgenic control, E9I9-MO injected embryos showed similar or slightly increased levels of Smyd1b_tv1^myc^ and Smyd1b_tv2^myc^ protein expression (Fig. 10A).

The subcellular localization of Smyd1b_tv1^myc^ or Smyd1b_tv2^myc^ was analyzed in the *smyd1b* knockdown embryos. The results showed that knockdown of endogenous *smyd1b* expression advanced the timing of Smyd1b_tv1 sarcomeric localization (Fig. 10C). Compared with the uninjected *smyd1b_tv1^myc^* embryos that showed little or no sarcomeric localization at 24 hpf (Fig. 10B), the E9I9-MO injected embryos showed a clear sarcomeric localization of Smyd1b_tv1^myc^ at 24 hpf (Fig. 10C). In contrast, knockdown of endogenous *smyd1b* had no effect on cytoplasmic localization of Smyd1b_tv2 (Fig. 10E). Similar to control embryos (Fig. 10D), Smyd1b_tv2 remained in cytoplasm of *smyd1b* knockdown embryos at 24 hpf (Fig. 10D, E). Together, these data indicate that knockdown of the endogenous *Smyd1b* expression could enhance the sarcomeric localization of Smyd1b_tv1^myc^ in zebrafish embryos.

### The Smyd1b-tv1 sarcomeric localization was disrupted in hsp90α1 mutant embryos

**Figure 11:**
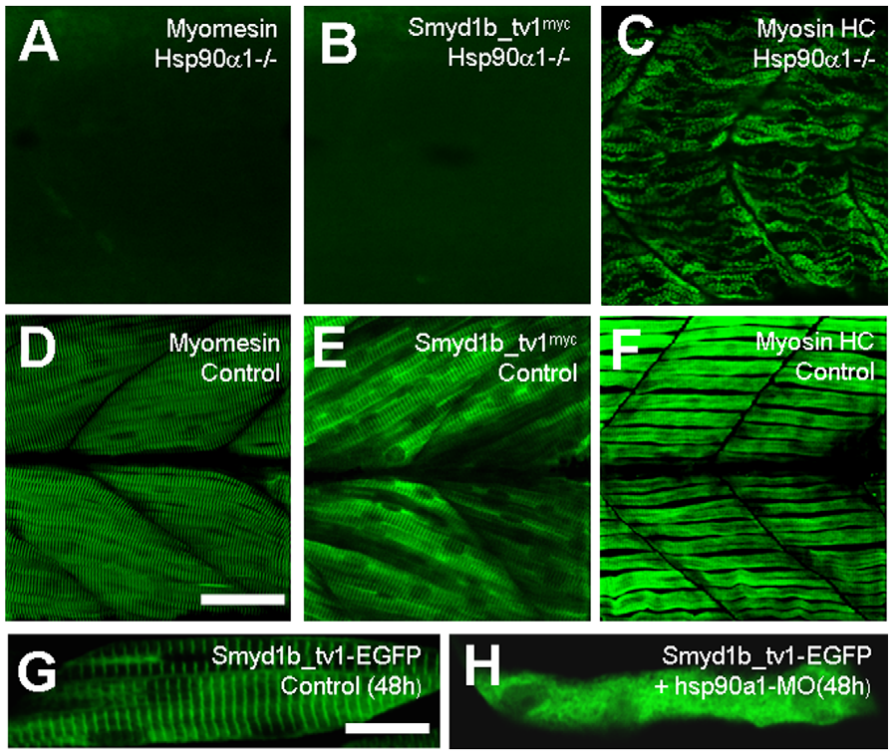
The effect of *hsp90α1* mutation or knockdown on Smyd1b_tv1 sarcomeric localization. **A and D.** Immunostaining using anti-myomesin antibody shows the organization of myomesin in *hsp90α1* mutant (A), or control (D) *smyd1b_tv1^myc^* transgenic embryos at 72 hpf. **B and E.** Immunostaining using anti-myc antibody shows the localization of Smyd1b_tv1^myc^ in *hsp90α1* mutant (B), or control (E) *smyd1b_tv1^myc^* transgenic embryos at 72 hpf. **C and F.** F59 staining shows the organization of slow muscle myosin in *hsp90α1* mutant (C), or control (F) *smyd1b_tv1^myc^* transgenic embryos at 72 hpf. **G and H.** DNA construct *Smyd1_tv1-EGFP* was injected alone or together with *hsp90α1* ATG-MO into zebrafish embryos. Smyd1_tv1-EGFP localization was determined in myofibers of the control (G), or *hsp90α1* knockdown (H) at 48 hpf. Scale bars: D = 40 µm; G = 15 µm.

It has been reported that *hsp90α1* knockdown or mutation severely disrupts the myofibril organization in skeletal muscles of zebrafish embryos [8]–[10]. To determine the effect of *hsp90α1* mutation on Smyd1b_tv1 sarcomeric localization, we generated Smyd1b_tv1^myc^ transgenic fish in the *hsp90α1* mutant background and characterized Smyd1b_tv1^myc^ localization in *hsp90α1* homozygous mutant Smyd1b_tv1^myc^ transgenic zebrafish embryos. The result showed that *hsp90α1* mutation which disrupts M-line organization (Fig. 11A) completely abolished the sarcomeric localization of Smyd1b_tv1^myc^ (Fig. 11B) despite some disorganized thick filaments remained (Fig. 11C).

To confirm the requirement of Hsp90α1 on Smyd1b_tv1 localization, we analyzed Smyd1b_tv1-EGFP localization in *hsp90α1* knockdown zebrafish embryos. Compared with the uninjected control (Fig. 11G), knockdown of *hsp90α1* completely blocked the sarcomeric localization of Smyd1b_tv1-EGFP in myofibers of zebrafish embryos (Fig. 11H). Collectively, these data indicate that the M-line structure is essential for the Smyd1b sarcomeric localization, reinforcing the idea that Smyd1b is localized on the M-line of sarcomeres.

## Discussion

In this study, we analyzed the subcellular localization of Smyd1b_tv1 and Smyd1b_tv2 during muscle development in zebrafish embryos. We found that Smyd1b_tv1 and Smyd1b_tv2 were primarily localized in the cytosol of myoblasts and myotubes in early stage embryos. However, they showed a sarcomeric localization in the differentiated myofibers of late stage zebrafish embryos and adult skeletal muscles. Compared with Smyd1b_tv2, Smyd1b_tv1 showed a stronger sarcomeric localization, and the sarcomeric localization is restricted to the M-line. The stronger sarcomeric localization of Smyd1b_tv1 requires Phe223 and Ser225 located within the Smyd1b_tv1 specific 13 aa insertion.

### Cytosolic localization of Smyd1b_tv1 and Smyd1b_tv2 in myoblasts

It has been reported that Smyd1 is localized in the nucleus of C2C12 myoblasts and undergoes a nucleus-to-cytoplasm translocation during myoblast differentiation *in vitro* [5]. In this study, we found that Smyd1b_tv1 and Smyd1b_tv2 were primarily localized in the cytosol of myoblasts and early myotubes of zebrafish embryos. Very little or no nuclear localization could be detected in myoblasts and myotubes of zebrafish embryos *in vivo*. The discrepancy between these two studies is not clear. It could be due to the use of two different model systems in these two studies, the *in vitro* C2C12 cell culture system versus the *in vivo* developing zebrafish embryos. C2C12 myoblast cells in culture may not fully resemble the process of muscle cell differentiation *in vivo*. It takes 1–2 days for C2C12 cells to differentiate *in vitro* whereas only a few hours are required for myoblast cell differentiation into myofibers *in vivo* in zebrafish embryos. It is possible that the nuclear localization is low and transient in developing muscles of zebrafish embryos that are below the sensitivity of detection by immunostaining. Consistent with this possibility, we found a weak nuclear localization when zebrafish Smyd1b_tv1 was expressed in C2C12 cells by DNA transfection (Data not shown). Moreover, the nuclear localization of zebrafish Smyd1b_tv1 was enhanced in C2C12 cells when incubated with a nuclear export blocker Leptomycin B (unpublished data). Collectively, these studies argue that Smyd1b_tv1 and Smyd1b_tv2 may exhibit a dynamic subcellular localization during muscle cell differentiation.

### Sarcomeric localization of Smyd1b_tv1 and Smyd1b_tv2 in myofibers

We showed that Smyd1b_tv1, and to some extent Smyd1b_tv2, were localized on the sarcomere of differentiated myofibers. Their sarcomeric localization appeared in a progressive fashion from anterior to posterior myotome during muscle development, correlating with the progression of myotome formation and muscle cell differentiation in zebrafish embryos. Double staining with sarcomere specific markers demonstrated that Smyd1b_tv1 was localized at the M-line. Disruption of M-line organization by Hsp90α1 knockdown or mutation completely abolished the sarcomeric localization of Smyd1b. Our findings are consistent with recent report by Just and colleagues showing the M-line localization of Smyd1b_tv1 in skeletal muscle fibers of zebrafish embryos [11]. Because the M-line is essential for stable contractions of the sarcomeres, the M-line localization of Smyd1b could be of biologically significant.

Interestingly, we showed that the M-line localization of myc-tagged Smyd1b_tv1 appeared earlier when the endogenous Smyd1b is knockdown. One possible explanation is that myc-tagged Smyd1b_tv1 proteins compete with the endogenous Smyd1b_tv1 proteins for the M-line localization. When endogenous Smyd1b_tv1 was knocked down, more myc-tagged Smyd1_tv1 proteins could be localized to the M-lines and thus become visible at an earlier stage of development. We do not expect to see a functional consequence of an advanced sarcomeric localization of the myc-tagged Smyd1b_tv1, because the myc-tagegd Smyd1b_tv1 could functionally replace the endogenous Smyd1b_tv1 [4].

Given that subcellular distribution of both Smyd1b splice forms is indeed different, what are the common functions of the isoforms since both isoforms are capable to rescue the loss of Smyd1b? It has been reported that Smyd1b_tv1 binds to myosin [11]. The myosin binding domain has been mapped to the C-terminal region between residues 278–390. This region is identical between Smyd1b_tv1 and _tv2, suggesting that both Smyd1b_tv1 and _tv2 are capable of binding to myosin, consistent with previous findings that both isoforms are capable to rescue the loss of Smyd1b [4].

Previous studies have indicated that Smyd1b is a histone methyltransferase which could methylate H3K9 in vitro [4]. Recent studies, however, have demonstrated that members of the Smyd family are able to methylate other non-histone proteins [12]. Protein methylation is a reversible post-translational modification, which controls biological activities and stability of proteins [13]–[15]. We speculate that Smyd1b could methylate structural or regulatory proteins at the M-line involved in sarcomere assembly and stability. Several muscle proteins including myosin, α-actin and creatine kinase are known to be methylated in skeletal muscles [16]–[18]. However, it remains to be determined whether any of them are the Smyd1 target proteins.

It should be noted that although sarcomeric localization of Smyd1b occurred after the α-actinin, α-actin, and MyHC, knockdown of Smyd1b completely abolished the sarcomere formation, thick and thin filament organization as well as Z-line and M-line in skeletal muscles of zebrafish embryos ([4], and unpublished data). These data suggest that Smyd1b is required for sarcomere assembly before their localization on the sarcomeres. However, it does not rule out the possibility that SmyD1b has additional function at the M-lines. It has been reported that myosin chaperones Unc45b and Hsp90α could be localized on sarcomeres of myofibers. Moreover, they shuttle between the A band and the Z line in response to stress or damage to the myofiber [19]. It has been suggested that the sarcomeric localization of myosin chaperones and their translocation within different parts of the muscle cells could be involved in the response of muscle cells to mount efficient physiological responses to muscle stress, load requirements, and/or stretch. It remains to be determined whether the sarcomeric localization of Smyd1b_tv1 is involved in sarcomere remodeling and response to muscle stress.

### Regulation of sarcomeric localization by Ser225 and Phe223

Although both Smyd1b_tv1 and Smyd1b_tv2 showed the sarcomeric localization, they differ significantly in timing and intensity of sarcomeric localization. Smyd1b_tv1 appeared earlier than Smyd1b_tv2 on the sarcomeres, and the sarcomeric localization is stronger than Smyd1b_tv2. Data from this study demonstrated that the enhanced sarcomeric localization of Smyd1b_tv1 requires Phe223 and Serine 225. Substitution of Phe223 or Ser225 with alanine significantly reduced its sarcomeric localization to a similar level as Smyd1b_tv2. Serine phosphorylation is a common post-translational modification involved in the regulation of protein subcellular localization. Phosphorylation of NFAT has been shown to play a vital role in its shuttling between the nucleus and sarcomeres [20]–[22]. Post-translation modification on Ser225 of Smyd1b_tv1 could be a potential mechanism regulating its sarcomeric localization. Consistent with the potential regulation by phosphorylation, substitution of Ser225 with threonine had no effect on its sarcomeric localization. However, substitution of Ser225 with aspartic acid, which mimics phosphorylation, significantly reduced Smyd1b_tv1 sarcomeric localization, arguing against the idea that phosphorylation of Ser225 is involved in regulation of Smyd1b_tv1 sarcomeric localization. The mechanism by which Ser225 is involved in Smyd1b_tv1 sarcomeric localization remains to be determined. In addition to being a potential phosphorylation site, Serine residues could also serve as target sites for O-glycosylation. However, Smyd1b appears to be an intracellular protein that makes it an unlikely target for O-glycosylation, suggesting that post-translational modification by other mechanisms may be involved in the increased sarcomeric localization of Smyd1b_tv1. Alternatively, the Phe223 and Ser225 might be part of translocation signal or protein motif required for the M-line localization. S225A or F223A substitution might result in a different protein shape masking or changing the translocation signal and lead to disruption of the M-line localization.

## Materials and Methods

### Zebrafish lines and maintenance

Mature zebrafish were raised at the zebrafish facility of the Aquaculture Research Center, Institute of Marine and Environmental Technology. The fish were maintained at 28°C with a photoperiod of 14 h light and 10 h dark, in 8-gallon aquaria supplied with freshwater and aeration. The *Tg(smyd1-smyd1b^myc^_tv1)mb6* and *Tg(smyd1-smyd1b^myc^_tv2)mb7* transgenic zebrafish lines were generated as described [4]. The minigenes were constructed using cDNA encoding the myc-tagged Smyd1b_tv1 or myc-tagged SmyD1b_tv2 cloned after the 5.3 kb muscle-specific zebrafish *smyd1b* promoter and its 5′-flanking sequence [23]. The *slo^tu44c^* mutant zebrafish line was obtained from Tubingen Zebrafish Stock Center. The *slo^tu44c^* mutant carries a nonsense mutation in the *hsp90α1* gene resulting in truncated molecules missing the C-terminal domain, which is important for both homo- and heterodimerization [10]. The *smyd1-smyd1b^myc^_tv1* transgenic fish was crossed with *slo^tu44c^* heterozygous mutant to generate *smyd1-smyd1b^myc^_tv1/slo^tu44c^* transgenic line. The myomesin-RFP zebrafish line (GBT0067) was obtained from Steve Ekker's laboratory at Mayo Clinic. It carries a RFP insertion in myomesin 3 [24]. Additional information can be found at http://www.zfishbook.org/index.php?topic=GBT0067#.

### Synthesis of morpholino-modified antisense oligos

The Smyd1b splicing blocker (E9I9-MO, 5′-CGTCACCTCTAGGTCTTTAGTGATG-3′) was based on the sequence of splicing site at the exon-9 and intron-9 junction of zebrafish *smyd1b* gene [4]. The *hsp90α1* translation blocker (ATG-MO, 5′- CGACTTCTCAGGCATCTTGCTGTGT- 3′) was targeted to sequence near the ATG start codon of zebrafish hsp90α1 mRNA transcript [9].

### Morpholino and DNA microinjection in zebrafish embryos

Morpholino antisense oligos were dissolved in 1×Danieau buffer [25] to a final concentration of 0.5 mM. DNA plasmid was dissolved in water at 50 ng/µl. 1–2 nl of MO or DNA was injected into zebrafish embryos at 1 or 2 cell stages. For morpholino and DNA co-injection, morpholino (1 mM) and DNA (100 ng/µl) were mixed at 1∶1 ratio and 1–2 nl of the mixture was injected into zebrafish embryos at 1 or 2 cell stages.

### Immunostaining of cryostat sections and whole mount zebrafish embryos

For immunostaining with cross sections, skeletal muscles were dissected from trunk muscles of transgenic zebrafish (90 dpf) expressing Smyd1b_tv1^myc^ and Smyd1b_tv2^myc^. The muscle tissues were fixed in 4% paraformaldehyde for 1 h at room temperature. The fixed samples were washed with 1×PBS-Tween (PBS, 0.1% Tween) 2×10 min, and then soaked in 30% sucrose for 2 h. The samples were transferred into an embedding chamber filled with OCT cryostat embedding medium (Tissue Tek). The embedding chambers were frozen on dry ice, and frozen blocks were cut on a cryostat at −20°C to produce 15 µm sagittal sections. Sections were transferred to subbed slides and allowed to dry completely at 37°C for 1 h. Sections were rehydrated in PBS-Tween, and non-specific staining was blocked using 10% goat serum in PBS-Tween for 10 min. Sections were then incubated overnight at 4°C in primary antibodies diluted in PBS-Tween. They were then washed with PBS-Tween for 5×5 min and incubated with fluorescence-labeled secondary antibodies, appropriate for the primary antibody isotype, for 1 h at room temperature. Sections were coverslipped in 50% Vector shield (Invitrogen) and observed under fluorescence microscopy (Axioplan-2, Zeiss).

Immunostaining with whole mount zebrafish embryos was carried out as previously described [4]. *T*g*(smyd1b_tv1)mb6* or *Tg(smyd1b_tv2)mb7* transgenic zebrafish embryos were fixed at 14, 24, 27, 30, 48 and 72 hpf. The subcellular localization of Smyd1b_tv1 or Smyd1b_tv2 in myoblasts, myotubes, and myofibers was determined by anti-myc antibody staining. The following antibodies were used for both cryostat sections and whole mount zebrafish embryos: anti-myc monoclonal antibody (9E10, DSHB), anti-myc polyclonal antibody (Cell Signaling Technology, #2272), anti-α-actinin (clone EA-53, #A7811, Sigma), anti-MHC for slow muscles (F59, DSHB), anti-myomesin (mMaC myomesin B4, DSHB). Secondary antibodies were FITC or TRITC-conjugates anti-mouse or anti- rabbit antibodies (Sigma).

### Mutagenesis

To generate Smyd1b_tv1 constructs with S217A, S225A, S225T, S225D, T221A, F223A, H224A and Q226A mutations, we carried out the mutagenesis using the QuikChange site-directed mutagenesis kit (Stratagene). The *smyd1-smyd1b^myc^_tv1* plasmid was used as DNA template. The following PCR primers were used.

S217A+T221A-f: 5′-AATCAGGCGGCCATCGATGCTGTfGTTT-3′

S217A+T221A-r: 5′-AAACACAGCATCGATGGCCGCCTGATT-3′

S225A-f: 5′-GTGTTTCACGCTCAGAAGAGG-3′

S225A-r: 5′-CCTCTTCTGAGCGTGAAACAC-3′

A225T-f: 5′-GATACTGTGTTTCACACTCAGAAGAGGATTG-3′

A225T-r: 5′-CAATCCTCTTCTGAGTGTGAAACACAGTATC-3′

S225D-f: 5′-ATACTGTGTTTCACGATCAGAAGAGGATTGA-3′

S225D-r: 5′-TCAATCCTCTTCTGATCGTGAAACACAGTAT-3′

F223A-f: 5′-CATCGATACTGTGGCTCACTCTCAGAAG-3′

F223A-r: 5′-CTTCTGAGAGTGAGCCACAGTATCGATG-3′

H224A-f: 5′-CGATACTGTGTTTGCCTCTCAGAAGAGG-3′

H224A-r: 5′-CCTCTTCTGAGAGGCAAACACAGTATCG-3′

Q226A-f: 5′-TGTGTTTCACTCTGCGAAGAGGATTGAG-3′

Q226A-r: 5′-CTCAATCCTCTTCGCAGAGTGAAACACA-3′

### Construction of Tol2-smyd1_tv1-EGFP, Tol2-smyd1_tv2-EGFP and their derived mutant constructs

pTol2-smyd1b_tv1-EGFP and pTol2-smyd1b_tv2-EGFP constructs were generated by cloning the smyd1b_tv1 and smyd1b_tv2 coding sequence in frame upstream of the EGFP coding sequence in the Tol2 vector. Briefly, the smyd1b_tv1 and smyd1b_tv2 coding sequence without the stop codon were generated by PCR using pfu DNA polymerase (Stratagene). DNA plasmid *smyd1-smyd1b^myc^_tv1* and *smyd1-smyd1b^myc^_tv2* were used as template amplified using the smyd1b-F (5′-CGGGATCCATGGAGTTTGTGGAAGTTTTTGA-3′) and smyd1b-R (5′-CGGGATCCTTCCTGCGGAACAGGTTCTTGAT-3′) primers. A *BamHI* site was introduced at the 5′ and 3′ ends of the smyd1b_tv1 or smyd1b_tv2 coding sequence via the PCR primers. The PCR products were digested with *BamHI* and then cloned into the *BamHI* site of the T2A200R150G vector [26]. The DNA sequence at the smyd1b and EGFP junction was confirmed by sequencing.

pTol2-smyd1b_tv1-EGFP(S225A), pTol2-smyd1b_tv1-EGFP(S225T), pTol2-smyd1b_tv1-EGFP(S225D), pTol2-smyd1b_tv1-EGFP(F223A), pTol2-smyd1b_tv1-EGFP(H224A), and pTol2-smyd1b_tv1-EGFP(Q226A) mutant constructs were generated by PCR using the QuikChange site-directed mutagenesis kit (Stratagene) as described previously. DNA plasmid pTol2-smyd1b_tv1-EGFP was used as template amplified using the S225A-f/r, S225T-f/r, S225D-f/r, F223A-f/r, H224A-f/r and Q226A-f/r primer sets described previously.

### Analysis of protein expression by Western blot

Western blot analysis was performed with control and *smyd1b* knockdown zebrafish embryos as described [27]. Chorions were removed from control, or MO-injected embryos (50 embryos for each group) at 24 hpf. Yolk sacs were removed by gently pipeting embryos through a glass pipet in 1 ml of PBS buffer. The embryos were collected by centrifugation at 5000 rpm for 20 seconds. The pellet of embryos was dissolved in 150 µl of 2× SDS loading buffer (3 µl for each embryo), and homogenized with a 23 gauge needle. 2 µl of PMSF was added to reduce the bubbles. The sample was boiled for 3 min at 100C. 20 µl of protein sample was analyzed on a 7.5% SDS PAGE. The proteins were transferred onto an Immobilon-P membrane (Millipore) and immunostaining was carried out using anti-myc (9E10; DSHB), and anti-γ-tubulin (T6557; Sigma) antibodies.
